# Impact of the COVID-19 pandemic on children's mental health: A systematic review

**DOI:** 10.3389/fpsyt.2022.975936

**Published:** 2022-10-18

**Authors:** Catalina Sau Man Ng, Sally Sui Ling Ng

**Affiliations:** Department of Early Childhood Education, The Education University of Hong Kong, Hong Kong, Hong Kong SAR, China

**Keywords:** COVID-19, mental health, internalizing behaviors, externalizing behaviors, children, systematic review

## Abstract

**Background:**

The outbreak of COVID-19 in December 2019 has caused unprecedented disruption to the structure of children's daily lives due to school closures, online learning, strict social distancing measures, limited access to outdoor activities and many other restrictions. Since children are more susceptible to stress than adults and there is a growing concern about the potential debilitating consequences of COVID-19 for children's mental health, the present review aims to provide empirical evidence on the groups who are most at risk of mental health problems and uncover the risk and protective factors of children's mental health.

**Methods:**

A systematic search was performed, in accordance with PRISMA guidelines, in the electronic databases Web of Science (including SSCI and A&HI) and EBSCOhost (including ERIC, MEDLINE and APA PsycArticles and APA PsycINFO), for any empirical studies published between January 2020 and February 2022 that focused on children ≤ 12 years old.

**Results:**

An initial search identified 2,133 studies. A total of 30 studies fulfilled the inclusion criteria and were analyzed. The evidence showed that many children were affected by the COVID-19 pandemic and experienced internalizing and externalizing behaviors. Worsened child mental health outcomes reflected socioeconomic inequalities as most at-risk children had parents with low educational attainment, were from families of low socioeconomic status and lived in small homes. Key risk factors were identified, including unhealthy lifestyle behaviors (extended screen time, sleep disturbances and less physical activity), increased pandemic-related stressors among parents and deteriorated mental health of parents, which were directly or indirectly associated with the pandemic safety measures, such as home confinement or social distancing. Protective factors including parents' resilience, positive parent-child relationship and school connectedness in relation to children's mental health were reported.

**Conclusion:**

The overall results highlight the urgent need for the implementation of tailor-made interventions for children with signs of internalizing and externalizing behaviors. Health promotion and prevention strategies by the government to maintain the mental health of children, particularly those from lower SES families who are at higher risk of worsened mental health are essential for post-pandemic policies.

## Introduction

The coronavirus disease of 2019 (COVID-19) has been a pandemic with destructive human, social and economic consequences since December 2019. In the absence of effective pharmaceutical interventions for the prevention and control of the novel coronavirus, stringent public health measures such as mask-wearing, strict social distancing, school and workplace closures as well as tough travel restrictions have been extensively implemented to mitigate the spread of COVID-19 (1, 2). Therefore, the pandemic has impacted people's daily lives with differential effects on various age groups (3).

Children are one of the groups who were unprecedently affected by the pandemic [i.e., temporary closure of daycare centers and schools, online homeschooling, limited access to recreational facilities and many other restrictions; (4–8)]. They experienced social isolation from peers, teachers, extended family and community which increases the risk of developing mental health issues such as anxiety and depression (9). In addition, with the disruption of their daily routine due to school closures, some children who are confined at home spend more time on using computers, iPhones or watching television but lack enough physical activities (10, 11). Prior studies found that excessive screen time negatively affected the cognitive and socio-emotional development of children (12, 13) and was associated with sleep disruption (14, 15) which aggravated the physical and psychological health of children (16).

As children have fewer personal resources than adults to cope with the sudden changes brought about by the pandemic (17), parents are the closest ones whom they turn to when unable to have direct contacts with other adults such as teachers and grandparents. However, during the pandemic, most parents have faced many challenges including financial difficulties (wages or salary reductions, job losses) and coping with parenting (working from home while taking care of children's homeschooling, managing their free time and dealing with their demands). Coupled with the parents' own needs but lacking sufficient support from either extended family, friends or other community organizations (18), high levels of parental stress can have a detrimental effect on children's physical and psychological health *via* parenting (19). Cheng et al. (18) found that parental stress predicted child abuse and neglect.

Since children's mental health is strongly related to their parents' mental health (20), with a recent study reporting that living with a parent with poor mental health increased the odds of poor child mental health (OR = 2.80, 95% CI 2.59–3.03) (21), parents are one of the key factors influencing the development of psychological problems in children (22). Given the increased stress and responsibilities of parents during the pandemic, children may have received insufficient adult support in their daily lives. The exposure to stressors can undermine children's neurobiological and socioemotional development (23). A study by the United Nations International Children's Emergency Fund (UNICEF) on 1,700 children and adolescents from 104 countries found that the brain development of children experiencing high levels of stress can be affected (24). With the escalating fear of contracting the disease and various risk factors, there was an increase in irritability, sleep disorders, anxiety and depression in children (7, 25–27).

Understanding how the pandemic undermines children's mental health is imperative as the effects can be long lasting (28). As argued by Henderson et al. (29), using Bronfenbrenner's ecological systems theory (EST) (30) can help better understand how the impact of the pandemic affects the mesosystem, exosystem, macrosystem and chronosystem which become risk factors for children's microsystem.

Till now, studies on children's mental health remain limited as most studies focus on either adolescents or adults (31, 32). Thus, designing suitable interventions to improve children's mental health becomes difficult without strong empirical evidence (33–35). This systematic review aimed to gather evidence on the current state of knowledge of the types of children who are at greater risk of mental health problems and the associated risk and protective factors for the pediatric population aged 12 years or below. Based on the timely evidence reported, the current review can inform policy makers, school administrators working in kindergartens and primary schools, researchers and healthcare providers and can prompt them to develop tailor-made interventions and devise relevant support programs to strengthen children's resources and help them cope with the risk factors that were identified in this review.

## Methods

### Search strategy

We searched the electronic databases of Web of Science and EBSCOhost including ERIC, MEDLINE, APA PsycArticles and APA PsycINFO, from January 2020 to February 2022. The Boolean operator was used in the search strategy, with “OR” and/or “AND” used to link search terms. The asterisk “^*^” was used as a wildcard symbol appended at the end of the terms to search for variations of those terms. We describe the complete search strategy below:

(a) “covid-19” OR “coronavirus” OR “2019-ncov” OR “SARS-CoV-2” OR “cov-19” OR “2019 pandemic” OR “pandemic.”
(b) “mental health” OR “mental illness^*^” OR “mental disorder^*^” OR “psychiatric illness^*^” OR “depress^*^” OR “anxiety.”
(c) “children^*^” OR “kids” OR “child^*^” OR “childhood.”
(d) a AND b AND c;
(e) Remove duplicates from d (if any);
(f) Limit f to “full-text” and “academic journal.”

In addition to electronic databases, the reference section of the included studies was hand-searched for additional relevant studies.

### Eligibility criteria

#### Inclusion and exclusion criteria

The articles included in this review are original studies published in peer-reviewed journals in English. The following inclusion criteria were applied: (i) mental health outcomes quantitatively or qualitatively measured; (ii) children aged 12 years or below; (iii) cross-sectional or longitudinal designs; (iv) original empirical data. The exclusion criteria include: (i) studies published not in English; (ii) children with pre-existing mental health condition (e.g., anxiety disorder or neurodevelopmental disorder) or disability (e.g., cerebral palsy), those who are homeless and those with substance abuse problems) as our objective is to understand the impact of the pandemic on children's mental health, so excluding children with pre-existing mental health/ health issues can provide us with clearer results; (iii) interventions; (iv) case reports, case studies, reviews, meta-analyses, opinions, editorials, commentaries, letters to the editor and conference abstracts.

#### Selection of sources of evidence

**Figure 1:**
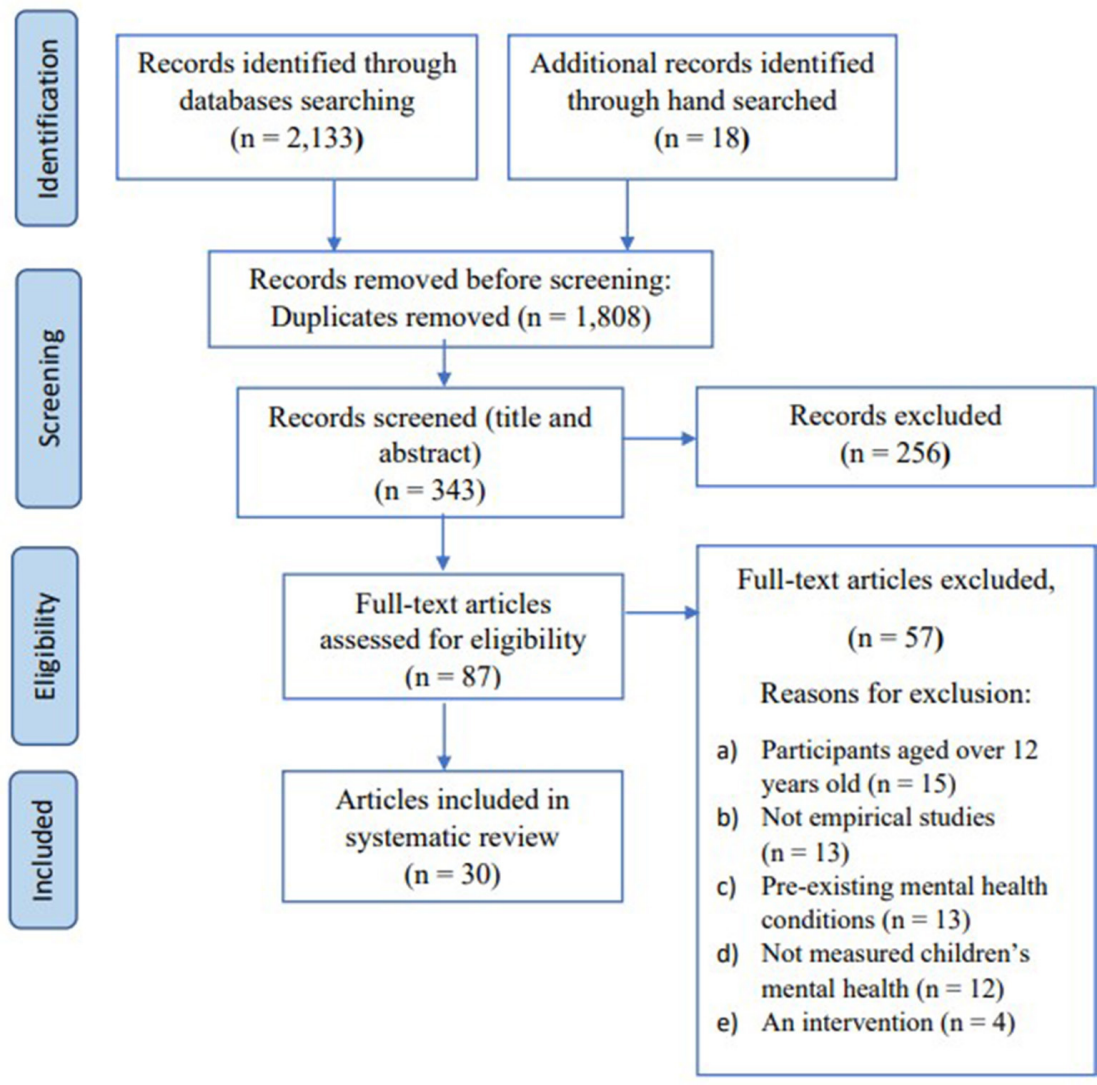
PRISMA flow chart outlining the study selection process.

The search of the Web of Science and EBSCOhost including ERIC, MEDLINE and APA PsycArticles and APA PsycINFO yielded a total of 2,133 records. Figure 1 presents the Preferred Reporting Items for Systematic Reviews and Meta-Analyses (PRISMA) flowchart describing the search process and the reasons for exclusion in this review (36). Both authors (CSMN and SSLN) first screened all the titles and abstracts independently. We discussed the disagreements and revised the selection. Then SSLN reviewed the full-text articles and 20% of the full-text articles assessed for eligibility were reviewed by CSMN. The inter-rater reliability was 85%. Any disagreements were resolved through discussion and consensus.

#### Quality assessment, analysis and data synthesis

All the included articles were assessed in accordance with a reported structured questionnaire and its criteria (i.e., good, fair, poor and very poor) with detailed descriptions of the ratings (37). The results were shown in Supplementary Table A1. All the included articles were extracted under a structured frame (38) (i.e., study design, sample size, the selection of participants, measurements, key findings, limitations and implications).

## Results

### Overview of included studies

A total of 30 original studies were identified, subsequently analyzed and summarized (see Tables 1, 2 for basic information, findings and limitations of the studies). The included empirical studies were conducted globally, including North America (Canada and U.S.A.), Europe (Denmark, France, Germany, Italy, Norway, Portugal, Sweden, U.K.,), Asia (China, India, Israel, Japan, and South Korea) and Oceania (Australia). Cross-sectional designs were used in 19 studies whereas 11 studies were longitudinal. Data were collected between February 2020 and July 2021 through phone or online surveys. The sample size varied significantly, from 40 to 21,526 participants. Most of the scales [e.g., Strengths and Difficulties Questionnaire (SDQ), Screen for Child Anxiety Related Disorders (SCARED), Child Behavioral Checklist (CBCL) and Children's Sleep Habits Questionnaire (CSHQ)] were validated or widely published in empirical studies on those topics.

**Table 1:** Basic information of the 30 included studies.

**Study** **No**.	**References**	**Country**	**Year of study**	**Study design**	**Participants**	**Child age** **M ±SD** **age range** **(AR)**	**% of boys (A)**	**Parent age** **M ±SD** **age range (AR)**	**% of** **mothers (B)**	**Mental health measures**	**Child mental health outcomes**
1	Bate et al. (39)	USA	31 Mar−15 May 20	C	158 parents	8.73 ± 2.01years AR: 6–12 years	56%	39.14 ± 5.96 years AR: NR	96%	CRIES, PSC	Int, Ext, H/I, PTSD
2	Bhogal et al. (40)	USA	T1: May 20 T2: Aug 20	L	64 parent-child dyads	8.2 ± 0.7 years AR: 7–10 years	37.5%	NR	NR	PSC, FIVE	Int, Ext, Fears of illness, Fears about social distancing
3	Browne et al. (41)	Canada	T1: Dec 19–Jan 20 T2: Feb 20 T3: Mar 20	L	231 educators	5.69 ± 2.09 years AR: 3–12 years	54.2%	NR	NR	IRS, SDQ	Int, Ext
4	Christner et al. (42)	Germany	End of Apr–Early May 20	C	2,672 parents	NR AR: 3–10 years	NR	NR	NR	SCQ on Child's strain (stress, irritability or loneliness); KIDSCREEN-52, SDQ	Child's strain, Int, Ext, H/I,
5	Di Giorgio et al. (43)	Italy	1–9 Apr 20	C	245 mothers	4.1 ± 0.92 years AR: 2–5 years	52%	37.31 ± 4.61 years AR: 23–49 years	100%	PSQI, BRIEF-P, SDQ-P, DERS	H/I, Ext, EF, ER, Sleep,
6	Dollberg et al. (44)	Israel	Mid Mar–end of Apr 20	C	140 mothers	4.17 ± 0.87 years AR: 3–6 years	50%	35.55 ± 4.59 years	100%	CBCL	Int, Ext
7	Dubois-Comtois et al. (45)	Canada	18 Apr – 18 May 20	C	144 parent-child dyads	10.44 ± 1.09 years AR: 9–12 years	51.4%	40.1 ± 5.11 years AR: 27–59 years	91%	12-item Negative Experienced Aloneness Scale, CBCL, FCV-19S, YSR	Anx, Int, Ext, Child aversion to aloneness
8	Foley et al. (46)	Australia, China, Italy, Sweden, UK, USA	1 Apr−7 Jul 20	C	2,516 parents	5.77 ± 1.1 years AR: 3–8 years	52.1%	37.15 ± 5.39 years, AR: 21–65 years	81.5%	SDQ	Int, Ext, H/I
9	Frigerio et al. (47)	Italy	T1 (pre-pandemic): child aged 1 year T2 (pre-pandemic): child aged 3 years T3 (Lockdown): child aged 4 years	L	T1: 94 mothers T2: 88 mothers T3: 59 mothers	4.2 ± 0.61 years AR: NR	54.2%	T3: Mother: 37.17 ± 3.51 years	100%	CBCL	Anx/Dep, Ext, H/I, Int, Sleep
10	Gassman-Pines et al. (48)	USA	20 Feb−27 Apr 20 23 Mar−26 Apr 20 (Sub-sample)	C	645 parents	4.9 ± 2.6 AR: 2–7 years	50%	31.0 ± 7.0 years AR: NR	83.1%	SCQ	Int, Daily child uncooperative behavior, Child sad or worry, Sleep
11	Giannotti et al. (49)	Italy	20 Apr−18 May 20	C	602 parents	AR: 3–11years	47.3%	Mothers: 40.58 ± 6.47 years Fathers: 41.96 ± 6.43 years	87%	SDQ	Ext
12	Hyunshik et al. (50)	Japan	T1 (Pre-COVID-19): Oct 19 T2 (During COVID-19): Oct 20	L	T1: 301 T2: 290 children	T1: 3.6 ± 0.3 years T2: 4.8 ± 0.3 years AR: 3–5 years	T1: 52.9% T2: 52.2%	NR	NR	SDQ, SCQ on sleep duration	Int, Ext, H/I, Sleep
13	Kerr et al. (51)	USA	Apr 20	C	1,000 parents	6.17 ± 3.67 years AR: 0–12 years		36.5 ± 6.0 years AR: 21–64 years	88.7%	PROPS National Survey of Children's Health	Stress, Positive behaviors
14	Köhler-Dauner et al. (52)	Germany	Jul 20	C	91 mothers	6.03 ± 0.61 years AR: 5–7 years	52.7%	38.14 ± 4.08 years AR: 31–46 years	100%	SDQ	Int, Ext, H/I
15	Köhler-Dauner et al. (53)	Germany	T1: child at 3 months T2: child at 12 months, T3: child at age 3 18 May−31 Jul 20	L	73 mothers	6.03 ± 0.61 years AR: 4.98–7.14 years	52%	38.20 ± 4.06 years AR: 31–46 years	100%	SDQ	Int, Ext, H/I
16	Larsen et al. (54)	Norway	T1 and T2: Dec 17–Jul 19 T3: 1 Apr−25 May 20	L	422 children	11.43 ± 2.59 years AR: 7–11	45%	NA	NA	SCARED, SMFQ, Emotional and Somatic/cognitive reactions scales	Anx, Dep, Emotional reaction, somatic/cognitive reactions, worry reactions
17	Li et al. (55)	China	15–29 Mar 20	C	21,526 parents	5.21 ± 1.4 years AR: 3–12 years	52.41%	NR	NR	CSHQ SDQ	Total difficulty (Int, Ext, H/I), Sleep
18	Liu et al. 2021 (56)	China	25 Feb−8 Mar 20	C	1,264 parent-child dyads	9.81 ± 1.44 years AR: 7–12 years	55.9%	NR	NR	SDQ	Int, Ext, H/I
19	Mariani Wigley et al. (57)	Italy	18 May−4 Jun 20	C	158 mothers	8.88 ± 1.41 years AR: 6–11 years	48.1%	43.27 ± 4.20 years AR: NR	100%	PMK-CYRM-R SCQ	Child's individual resilience Stress-related behaviors include ✓ Difficulty standing still ✓ Concentration difficulties ✓ Nervousness and irritability ✓ Tendency to cry without reasons ✓ Difficulty in sleeping ✓ Restless sleep with frequent waking ✓ Refuse to eat ✓ Excessive food seeking
20	McArthur et al. (58)	Canada	T1: 2017–19 T2: May–Jul 20 T3: Jul–Aug 20	L	846 mother-child dyads	9.85 ± 0.78 years AR: 9–11 years	52.8%	NR	100%	BASC-3 MDI SCQ on duration of sleep	Anx, Dep, Subjective wellbeing, Sleep
21	Moore et al. (59)	Wales, UK	T1: Feb–June 19 T2: Apr–Jul 21	L	4,032 children	NR AR: 10–11 years	47%	–	–	MMSQ	Int, Ext
22	Moulin et al. (32)	France	24 Mar−28 Apr 20	C*	432 parents	6.8 ± 4.10 years AR: NR	51%	NR AR: 27–46 years	65%	SDQ, SCQ on sleep difficulties	Int, Ext, Sleep
23	Oliveira et al. (60)	Portugal	middle of Jun–end of Jul 20	C	110 parent-child dyads	9.09 ± 0.80 years AR: 7–11 years	50%	NR	85%	KIDSCREEN-27, SDQ	Int, Ext Health-related quality of life
24	Park et al. (61)	South Korea	24–28 May 20	C	288 parents	5.56 ± 3.31 years AR: 1–12 years	NR	NR	92%	SCQ	Anx, Dep, Loneliness, Stress, Physical and psychological health, Sleep
25	Robertson et al. (62)	USA	T1: Apr 20 T2: Jun 20 T3: Jul 20	L	286 caregivers	6.21 ± 4.93 years AR: 1–7 years	NR	34.31 ± 6.68 years AR: 18–54 years	79.4%	SDQ	Int, Ext
26	Sama et al. (63)	India	2020	C	310 parents	NR	57.7%	NR	NR	SCQ	Anx, dep, anger, irritation, Diet, Weight, Sleep, Quarrels, Frequency of illness
27	Specht et al. (64)	Denmark	T1: 20 Feb−11 Mar 20 T2: Apr 20	L	40 parents	5.0 ± 0.7 years AR: 3.5–6.8 years	45%	NR	82%	SDQ	Int, Ext, H/I
28	Thompson et al. (65)	USA	T1: Apr 20 T2: Oct 20	L	147 mothers	21.35 ± 8.47 months AR: 7.76–37.18 months	48%	26.93 ± 6.15 years AR: 18–43 years	100%	CBCL	Int, Ext
29	Wang et al. (66)	China	26 Jun−6 Jul 20	C	6,017 caregivers	NR AR: NR	54.6%	NR	NR	SDQ	Total difficulties (Int, Ext, H/I)
30	Wang et al. (67)	China	15–29 Mar 20	C	16,398 parents	4.69 ± 0.75 years AR: 3–6 years	51.9%	NR	NR	SDQ, CSHQ,	Int, Ext, H/I, Sleep

Child age, mean age ± SD; AR, age range; (A) represents the percentage of boys; (B) represents the percentage of mothers; L, Longitudinal study design; C, Cross-sectional study design; NR, Not reported; “-”, not included in the study; C^*^, data collected from a longitudinal study; Anx, Anxiety; Dep, Depressive symptoms; Diet, Diet affected; Weight, Weight changed; EF, Executive functioning; ER, Emotion regulation; Int, Internalizing symptoms; Ext, Externalizing symptoms; H/I, Hyperactivity/Inattention; MH, Mental health; PTSD, Post-traumatic stress disorder; Sleep, Sleep quality.

BASC-3, Behavior Assessment System for Children; BRIEF-P, Behavior Rating Inventory of Executive Functions-Preschool version; CBCL, The Child Behavioral Checklist; CRIES, Child Revised Impact of Events Scale-13; CSHQ, Children's Sleep Habits Questionnaire; DERS, Difficulties in Emotion Regulation; FCV-19S, 7-item Fear of COVID-19 Scales 19S; FIVE, Fear of Illness and Virus Evaluation; IRS, Impairment Rating Scale; KIDSCREEN-27, Health-Related Quality of Life Questionnaire for Children and Adolescents 27-items; KIDSCREEN-52, Health-Related Quality of Life Questionnaire for Children and Adolescents 52-items; MDI, Middle Years Development Instrument; MMSQ, Me and My School Questionnaire; PMK-CYRM-R, Person Most Knowledgeable version of the Child and Youth Resilience Measure-Revised; PROPS, Parent-Report of Post-Traumatic Stress; PSC, 35-item Pediatric Symptom Checklist; PSQI, Pittsburgh Sleep Quality Index; SCARED, Screen for Child Anxiety Related Disorders; SCQ, Self-constructed questionnaire; SDQ, Strengths and Difficulties Questionnaire; SDQ-P, Strengths and Difficulties Questionnaire–Parent version; SMFQ, Short Mood and Feelings Questionnaire; YSR, Youth Self-Report.

**Table 2:** The key findings and acknowledged limitations of included studies.

**Study No**.	**References**	**Key findings extracted from included study**	**Acknowledged limitations**
1	Bate et al. (39)	Positive parent-child relationship was a significant moderator between parents' and children's emotional health during the COVID-19 pandemic. Parents with lower education level, younger children and lower household income reported to have higher anxiety and depressive symptoms. Parental mental health was adversely influenced by COVID-19 impact.	The study was cross-sectional and correlational so it did not establish any causality regarding the relationship between parents' and children's emotional health. The samples were mostly White, middle-class and healthy individuals so the results cannot be generalizable to other populations. The study used self-report questionnaires which were subject to biased reporting.
2	Bhogal et al. (40)	When compared to children with higher SES, lower SES children reported more fears about social distancing. In addition, children's fear of illness increased over time during the pandemic which was independent of SES and race. Lower SES children reported more internalizing problems at baseline but decreased following home confinement.	The survey relied on surveys which was subject to biased reporting. With small sample size and most participants being Black Americans, the results cannot be generalizable to other populations.
3	Browne et al. (41)	Male children studying in early childhood education showed increased mental health problems before the announcement of the outbreak of the pandemic. After the announcement, their mental health problems deteriorated significantly. No differences over time were observed for female children.	The study had small sample size. The data were collected from childcare workers which may be subject to reporter bias. The two time points for data collection were close which could not study the long-term effects of the pandemic. The study did not collect data on the socioeconomic status of families.
4	Christner et al. (42)	Both children (>50%) and parents (31%) experienced high level of stress during the lockdown. Older children aged 7-10 years experienced more emotional symptoms but less conduct problems and hyperactivity than younger children aged 3–6 years. The level of stress was influenced by social isolation and housing conditions. More older children reported that they had more problems with their homework and they lacked sports and hobbies during the pandemic. Children from single-parent families showed more emotional symptoms. Only child showed more emotional symptoms and hyperactivity/inattention. Externalizing behaviors in children were associated with housing conditions.	The study used parental report which was subject to reporter bias. Since the study used online survey, there may be sample bias as only the participants who were accessible to good internet and technical devices joined the study. Cross-sectional study design did not allow to detect the long-lasting effects of the pandemic.
5	Di Giorgio et al. (43)	Worsening sleep quality predicted increasing emotional symptoms and self-regulation difficulties in both children and mothers. Mothers' trait emotional fatigue was associated with children's ability to control their behavior. During home confinement, parental time pressure, limited space for children to discharge energy and disruption of daily routines were the risk factors which contributed to negative children's psychological outcomes.	The small sample size reduced the statistical power. The sample was not representative. The study used retrospective questions to compare the current situation to a baseline before the outbreak which were subject to biases.
6	Dollberg et al. (44)	Mothers who experienced more anxiety symptoms perceived more children's externalizing and internalizing behaviors. Maternal anxiety symptom was a mediator of children's behaviors during the pandemic. Mothers with higher mentalization skills, i.e., higher mind-mindedness, weakened the indirect effect of anxiety on the link between COVID-19 and children's externalizing behaviors.	The limitations included a small sample size, concurrent data collection in each group and a reliance on mothers as the only source of information.
7	Dubois-Comtois et al. (45)	Family factors such as parent-child relationship, parents' mental health and family dysfunction and chaos were strongly associated with children's psychological functioning. Greater depressive symptoms in parents and lower attachment security to parents were associated with higher internalizing problems in children. More family dysfunction and chaos and poor parent-child relationship predicted more externalizing behavior problems. Children's anxiety toward COVID-19 was associated with parents' anxiety of the pandemic and more child aversion to aloneness.	The design of the study was cross-sectional and no direction of effects could be made. The sample size was small. It might be likely that children answered the questions with the presence of family members so their responses might be influenced.
8	Foley et al. (46)	Familial risk factors such as parental distress, poor parent-child relationship and chaotic household predicted greater hyperactivity, emotional and conduct problems in children. Parental distress mediated the relationship between social disruption by the pandemic and child difficulties.	The convenience sample of parents from middle to high SES limited the generalizability of the findings. The reliance on online research designs during the pandemic increased selection bias and collider bias. Cross-sectional study design cannot be used to infer causality. Single informant might subject to reporter biases.
9	Frigerio et al. (47)	Children's internalizing and externalizing behaviors including emotional reaction, anxious-depression, withdrawal and aggression in preschool children were significantly increased during the lockdown. Greater maternal mood symptoms were significantly associated with the increase in children's internalizing and externalizing behaviors. Increased caregiving responsibilities due to the closure of childcare facilities triggered parenting hardships which adversely impacted maternal mood.	The study recruited a relatively small sample of mother-child dyads. Both maternal mood symptoms and children's problems were reported by mothers. The research design of the study did not allow to infer any causality of the association between maternal and children's wellbeing. Paternal mental wellbeing was not included in the analysis.
10	Gassman-Pines et al. (48)	Parents of vulnerable families reported higher frequency of daily negative mood since the outbreak of the pandemic. Parents' psychological wellbeing decreased during the post-COIVID-19 restrictions period. Parents' daily negative mood was significantly more frequent during post- than pre-restrictions. Those who experienced COVID-19-related hardships including unemployment, household income declines, caregiving burden and illness reported worse psychological wellbeing. Both caregiving burden and household illness were significantly associated with children's uncooperative behavior and worry.	The sample was limited to a vulnerable group of the population which was impacted by the pandemic. Therefore, the generalizability of the findings was limited.
11	Giannotti et al. (49)	There was higher level of parental stress particularly in mothers. There were heightened externalizing behaviors in children particularly younger boys during home confinement. Coparenting, being a mother, having younger child, less time to take care of the child and less feasibility to work remotely predicted parental stress. Child externalizing behaviors were predicted by gender (male), higher level of parental stress, less time devoted by parents to the child and the workload due to online learning.	Imbalanced sample size with more mothers compared to fathers. The participation in the study may be limited to parents who owned digital device and used social media. Cross-sectional study design did not allow us to understand the prolonged effects after the home confinement.
12	Hyunshik et al. (50)	Physical activities, recommended screen time and prosocial behaviors decreased significantly. There was increased sedentary time and hyperactivity-inattention behaviors.	The study was targeted a specific age group and region in Japan so it limited generalization. Parents completed the questionnaires which might subject to reporter biases.
13	Kerr et al. (51)	Parents perceived more psychological impacts from the pandemic reported higher levels of parental burnout, more children's stress behaviors and less positive behavior in children. Family income moderated the relationship between parents' psychological impacts and children's stress behaviors.	The convenience sample limits the generalizability of the findings. The study was cross-sectional so it did not infer any causality. Reporter bias existed as the child behavior questions were retrospectively reported by parents.
14	Köhler-Dauner et al. (52)	Maternal depression significantly and fully mediated the relationship between maternal attachment representations and children's mental health during the pandemic. However, the indirect effect of maternal attachment representations on children's mental health before the pandemic through maternal depression did not reach any statistically significance.	The sample was limited to mothers of the birth cohort of mother-child dyads. Mothers retrospectively provided their responses regarding their own depressive symptoms and their children's mental health which might be affected by social desirability and their memory. The study consisted of a small sample size.
15	Köhler-Dauner et al. (53)	A high level of mothers' perceived daily stress during the pandemic was found. Children's hyperactivity level during the pandemic was closely associated with mothers' perceived daily stress before the pandemic. However, there was no significant relationship between mothers' perceived daily stress and children's behavioral problems.	The study consisted of small sample size and the samples were not representative. Retrospect and self-report bias existed.
16	Larsen et al. (54)	Home school experience, child perceived family stress and instability, missing friends and worry about virus infection were significantly associated with all three outcomes (children's emotional, somatic/cognitive and worry reactions). Family stress and instability were found to be the strongest predictors. Older children were found more adversely impacted during the pandemic.	Retrospective questionnaires might be subject to recall bias.
17	Li et al. (55)	Parental education, sleep disturbance, less physical activity, higher media exposure, non-parental care, poor parental mental health and harsh parenting were independently associated with increased child mental health problems regardless of SES.	Data collection was restricted to the participants with internet access only. The study used cross-sectional design which could not reflect a long-term impact of the pandemic on socioeconomic inequality. Parents self-rated their own mental health which may subject to bias and social desirability.
18	Liu et al. (56)	Home quarantine during COVID-19 pandemic increased behavioral problems and emotional symptoms in school-aged children. Children with physical activity had a lower hyperactivity-inattention risk and less prosocial behaviors problems. Parental anxiety was associated with increased risks of emotional symptoms and total difficulty in children.	The study used cross-sectional design which failed to infer causality. The potential self-selection bias in the study should be noted.
19	Mariani Wigley et al. (57)	The COPEWithME positively correlated with mothers' and children's resilience. The relationship between mothers' resilience and children's stress behaviors was mediated by the ability of mothers to support and promote child resilient behaviors in school-age children.	The study has the following limitations: cross-sectional design, convenient sampling, small sample size and no paternal responses.
20	McArthur et al. (58)	Connection with caregivers (parent-child relationship), child sleep and their screen time were significant predictors of anxiety symptoms. On the other hand, connection with caregivers and screen time predicted depression. Connection with caregivers significantly predicted child happiness.	Selection bias existed because only mothers residing in an urban setting which was a portion of the cohort were recruited. Some child-reported variables were measured cross-sectionally.
21	Moore et al. (59)	When compared to pre-pandemic, elevated emotional difficulties among children were observed during the pandemic. The prevalence of emotional difficulties among girls was higher than boys. Children reported a high degree of school connectedness prior to and after the pandemic. Better teacher-pupil relationship predicted better mental health and life satisfaction. The positive feelings about the transition to secondary schools among children remained unchanged.	Cross-sectional study design could not detect the cause-and-effect direction. Significant cultural and social differences between countries may limit the generalizability of the findings.
22	Moulin et al. (32)	Children's psychological difficulties were associated with family financial difficulties, parental symptoms of anxiety and depression and the disruption of daily routine (i.e., children's sleeping difficulties and higher screen time exposure).	All measures were based on parents' self-report which was subject to bias and social desirability. There was no data on children's emotional difficulties and hyperactivity/inattention prior to the pandemic.
23	Oliveira et al. (60)	The lifestyles of children, in particular socioeconomically disadvantaged children, were characterized by a higher prevalence of sedentary behaviors (screen time) and fewer active leisure and playing activities. When compared with boys, girls engaged more in play and social activities, not physical activities. Protective factors such as regular sleep, active leisure, playing and learning activities and positive family coping strategies were linked to better child wellbeing.	The study used cross-sectional design which could not infer causality. The sampling did not allow for the generalization of the findings. Missing values, social desirability and recall bias (i.e., self-report) may have influenced the findings.
24	Park et al. (61)	The caregivers' childcare time increased significantly during the pandemic. For children, they spent significantly more time on online interactions and screen while time for face-to-face interactions and learning decreased significantly. The stress levels of both parents and children increased significantly during the pandemic.	Data were based on parents' self-report which was subject to recall bias and social desirability. The survey consisted of limited variables for the analysis. The sample was not representative as the online survey only reached the parents with access to the internet.
25	Robertson et al. (62)	Poor caregiver mental health at Time 1 predicted increased pandemic-related stress in caregivers at Time 2. Caregiver pandemic-related stress at Time 1 predicted the increase in internalizing problems in children at Time 2, which increased caregiver pandemic-related stress at Time 3. Poor mental health of caregivers at Time 2 was a predictor of increased child externalizing behaviors at Time 3.	The study did not use a standardized measure of resilience or measures of other risk factors which may affect the relationship between constructs. The study may not engage the vulnerable families which were most impacted by COVID-19 but may not have joined the study. The samples were from a large south-eastern city in the USA so the findings cannot generalize to other populations.
26	Sama et al. (63)	There was an increase in emotional problems (irritation, anger, anxiety and depression) in children during COVID-19. The correlation analyses showed that children's mental health was significantly related to increased screen time, sleep disorder, reduced outdoor activities, the area of their house, the number of children in the family, maternal education qualification and socio-economic status of their family.	The study did not include limitations.
27	Specht et al. (64)	There was a change of emotional and behavioral functioning in children during COVID-19. Those children who had leisure time activities prior to lockdown showed greater changes in functioning than those who did not have. An increased externalizing behavior in children was found. The decline in mental wellbeing in children was potentially due to parental stress and other risk factors such as the loss of private space, room for active play and the socialization with peers.	The study used a small sample size which made it impossible to conduct interaction analysis across the sample. The follow-up was short (only 3 weeks). In addition, it is probable that parents remembered their previous answers at baseline which could have affected the results.
28	Thompson et al. (65)	COVID-19 health risk and contextual hardships worsen maternal mental health significantly. Deteriorated maternal mental health at COVID-T1 predicted children's adjustment problems, and the concurrent children's adjustment problems at COVID-T2 were predicted by the COVID-19 contextual hardship and changes in maternal mental health. Poor maternal resilience in coping with COVID-19 health risk and hardships was related to increased children's adjustment problems.	The samples were mothers with young children who lived in low income contexts so the samples limited the generalizability of findings to other populations. The study did not examine a broader set of resilience factor before the pandemic and so, some of the resilience factors were assessed together with mental health. This might have been biased by mothers' mental health status. The study did not include second caregivers to understand maternal and child adjustment during the pandemic.
29	Wang et al. (66)	Children whose caregivers with low education level, were from low SES families and male tended to have more emotional and behavioral problems. The COVID-19-related knowledge and precautions predicted lower emotional and behavioral problems among children and the relationship was partially explained by the emotional problems in caregivers.	The study was cross-sectional so it cannot infer causality. A self-report online survey was not representative as it restricted to those who could access the internet. COVID-19-related knowledge and precaution was assessed by a single-item scale.
30	Wang et al. (67)	A higher parental wellbeing index was associated with lower child mental health problems. Harsh parenting and child sleep problems significantly mediated the relationship between parental wellbeing and child mental health.	The data cannot differentiate the findings between urban and suburban areas. Several variables were assessed by one or two self-constructed items. Some information was retrospectively reported by parents who might be biased. Cross-sectional study design did not infer the direction of causality.

### Study participants

Although children's mental health is one of the objectives of the current review, children were asked to complete surveys in three included studies only (50, 54, 59). The participants of the 30 included studies were mostly parents except Browne et al.'s study (41) which invited educators to assess children's mental health.

Children's mental health and other associated factors were mainly reported by parents. Among parents, mothers aged between 18 and 65 years were the main participants. Mothers were even the sole participants in seven included studies (43, 44, 47, 52, 53, 57, 65). Compared with mothers, the fathers' participation in research studies was low, so fathers were one of the respondents in ten included studies only [e.g., (32, 39, 45, 46, 48, 49, 51, 60–62, 64)]. Studies using both parent-child dyads as informants were also dearth (40, 45, 56, 58, 60).

### Summary of the included studies

#### Children's mental health during COVID-19

The included studies assessed children's mental health by measuring the levels of internalizing (stress, anxiety, depression, anger, irritation, withdrawal, trauma-related symptoms) and externalizing behaviors (aggressive behaviors, hyperactivity/inattention problems, conduct problems) and the level of prosocial behavior. Based on the findings of the included studies, children's mental health was generally on the decline. Children exhibited more stress-related behaviors, anger, irritability, withdrawal symptoms, fear and anxiety (of COVID-19) and higher levels of depressive symptoms. Using repeated cross-sectional surveys before and after the onset of the pandemic, Moore et al. (59) examined the changes in mental health difficulties, life satisfaction, school connectedness and feelings about the transition to secondary school among children aged between 10 and 11 in Wales. The results showed that emotional difficulties increased from 17% in 2019 (prior to the pandemic) to 27% in 2021 among children (OR 1.65; 95% CI 1.23–2.20). Based on 432 parent participants, Moulin El-Aarbaoui et al. (32) reported that 7.2% of French children showed signs of emotional difficulties. In a large population-based study (*n* = 21,526) by Li et al. (55), 32.3% indicated mental health problems. Sama et al. (63) reported alarmingly high figures - 73.15 and 51.25% of Indian children showed signs of increased irritability and anger, respectively. Christner et al. (42) showed that over 50% of German children reported to be rather or clearly stressed, irritated or lonely during the lockdown.

Apart from internalizing behaviors, children also displayed more externalizing behaviors during the pandemic. Liu et al. (56) reported that the prevalence of behavioral problems among school-aged Chinese children varied from 4.7 to 10.3% while in home quarantine during the pandemic. Moulin El-Aarbaoui et al. (32) found that 24.8% had symptoms of hyperactivity/inattention. Table 3 summarizes the prevalence of mental health problems among children in the included studies. Overall, the results of the included studies consistently point to a decline in child wellbeing globally.

**Table 3:** Prevalence of children's mental health problems during the COVID-19 pandemic based on the 30 included studies.

**Study** **No**.	**References**	**Prevalence of children's mental health problems**						**Stress**	**Anx**.	**Dep**.	**Int**.	**Ext**.	**H / I**	**PTSD**
1	Bate et al. (39)	Hyperactivity: 18.4%									✓	✓	✓	✓
		Internalizing behaviors: 20.3%												
		Externalizing behaviors: 18.4%												
		PTSD: 15.8%												
2	Bhogal et al. (40)	Fears of getting illness: 60%							✓					
4	Christner et al. (42)	Feeling stressed, irritated or lonely: 59%						✓						
5	Di Giorgio et al. (43)	Self-control Difficulties:										✓		
		(Before lockdown) 14.29%												
		(During lockdown) 21.23%												
10	Gassman-Pines et al. (48)	Child daily sad or worried									✓	✓		
		(Pre-COVID-19 restrictions): 22.5%												
		(Post-COVID-19 restrictions): 24.1%												
		Child daily uncooperative behavior:												
		(Pre-COVID-19 restrictions): 41.7%												
		(Post-COVID-19 restrictions): 45.1%												
17	Li et al. (55)	Total difficulty (SDQ): 32.31%									✓	✓	✓	
		Male: 33.67% vs. Female: 30.82%												
18	Liu et al. (56)	Internalizing behavior: 6.3%									✓	✓	✓	
		Externalizing behavior: 4.7%												
		Peer problems: 6.6%												
		Total difficulty (SDQ): 8.2%												
20	McArthur et al. (58)	Anxiety symptoms: 13.8%							✓	✓				
		Depressive symptoms: 8.2%												
21	Moore et al. (59)	Emotional difficulties:									✓	✓		
		2019: 17.5% vs. 2021: 27.4%												
		Boys: 2019: 14.4% vs. 2021: 21.6%												
		Girls: 2019: 20.3% vs. 2021: 29.5%												
		Behavioral difficulties:												
		2019: 13.3% vs. 2021: 14.6%												
22	Moulin El-Aarbaoui et al. (32)	Emotional difficulties: 7.2%,									✓		✓	
		Hyperactivity / Inattention: 24.8%												
24	Park et al. (61)	Nervousness: 17.4%						✓	✓	✓	✓			
		Anxiety: 2.1%												
		Depression: 2.1%												
		Loneliness due to limited social interactions: 46.9%												
25	Robertson et al. (62)	Internalizing behavior:									✓	✓		
		T1: 63.6% vs. T2: 37.4% vs. T3:43.7%												
		Externalizing behavior:												
		T1:63.3% vs. T2: 37.4% vs. T3: 43.7%												
26	Sama et al. (63)		Overall	Ludhiana	Sangrur	Ferozepur	Sangrur		✓	✓	✓	✓	✓	
		Anxiety	21.3%	21.4%	23.5%	12.2%	28.8%							
		Depression	20.9%	22.9%	21.2%	11.0%	30.1%							
		Quarrels	40.0%	70.0%	68.0%	63.5%	77.0%							
		Signs of irritability	73.15%	85.7%	68.2%	63.4%	75.3%							
		Anger	51.25%	80.0%;	58.8%	53.7%	75.3%							
29	Wang et al. (66)	Emotional and behavioral problems (EBPs)									✓	✓	✓	
		Shanghai and Taizhou: 12.5%												
		✓ SDQ total difficulties (slightly raised): 7.2%												
		✓ SDQ total difficulties (high and very high): 5.3%												

Anx., anxiety; Dep., Depressive symptoms; Int., Internalizing behaviors; Ext, Externalizing behaviors; H/I, Hyperactivity/Inattention; PTSD, Post-traumatic stress disorder.

#### Types of children at greater risk of negative mental health outcomes

The results from the included studies demonstrate that there are some children at greater risk of internalizing or externalizing behaviors. Those children are: (1) only child in the family; (2) from parents with low education qualifications; (3) from families of low socioeconomic status (SES); and (4) from a small size home.

Being the only child in the family puts a child at higher risk of mental health problems. Christner et al. (42) revealed that children without siblings experienced more emotional symptoms and hyperactivity/inattention than children with siblings. Sama et al. (63) found that there was a positive correlation between children's mental health (anxiety, depression, anger and irritation) and the number of children in the family (*r* = 0.04). Since Pearson's correlation was used, which merely provides an indication that there was a relationship between the two variables, and the *p-* value was not reported, whether the number of children in the family was a predictor of poor mental health for children requires further investigation.

Children of parents with low educational qualifications are also at greater risk of mental health issues. Four of the included studies observed significant links between parental education and children's mental health problems [e.g., (40, 48, 55, 66)]. Bhogal et al. (40) found that children of parents with lower educational qualifications were at higher risk of mental health difficulties by 40%. Li et al. (55) highlighted those children whose parents had low educational qualifications such as middle school or below had a 6% higher risk of mental health difficulties than those whose parents graduated from university. Similar findings were reported by Wang et al. (66) showing a higher prevalence of emotional and behavioral problems in children of caregivers with a lower educational level.

Children of low SES were also burdened by the effects of the pandemic. Bhogal et al. (40) found that children with low SES reported more fears about social distancing than their counterparts with higher SES. Moore et al. (59) found that children from low SES families reported an increase in emotional difficulties from 19.5 to 33.8%, compared to those from more affluent families who reported an increase from 11.7 to 18.5%. Li et al. (55) reported that Chinese children of low SES reported more mental health problems.

Sama et al. (63) found that the size of a child's home was associated with the mental health of children. It is likely that children in cramped living conditions are at greater risk of emotional and behavioral problems in cramped living conditions which may intensify conflicts among siblings or family members resulting in higher levels of psychological stress. Christner et al. (42) found that children living in an apartment reported higher hyperactivity/inattention (M = 4.38, SD = 2.33) than those with a large garden at home (M = 3.93, SD = 2.27).

#### Risk factors for children's mental health

Identifying the risk and protective factors is essential to understand why an unprecedented situation such as the COVID-19 pandemic is detrimental to children's mental health (40, 53, 65). Based on the 30 included studies, we identified the following risk factors:

#### Unhealthy lifestyle factors

##### Extended screen time

The temporary closure of schools and home confinement to mitigate the spread of COVID-19 translated to a sedentary lifestyle for children, including increased use of screen time and online interactions whereas face-to-face interaction time decreased significantly (68). Based on the cross-sectional data from Portuguese children studying in 3rd and 4th grade and their parents, Oliveira et al. (60) found that there was a higher prevalence of sedentary behaviors including TV and gaming/internet, i.e., activities that required a higher amount of screen time, particularly among socioeconomically vulnerable children. There were gender differences in screen time, with girls spending more time on watching TV and socializing online. Children from families with a negative socioeconomic change spent more time on watching TV and gaming/internet but less time on sleeping. Li et al. (55) assessed Chinese children's mental health problems in relation to factors including socioeconomic inequalities, lifestyle and family environment factors and found that media exposure (≥2 h per day) was independently associated with child mental health problems, regardless of SES.

Based on 846 mother-child dyads, McArthur et al. (58) reported that screen time predicted anxiety and depression in Canadian children. Those who engaged in excessive screen time reported higher levels of anxiety (Beta = 0.11; 95% CI 0.04–0.17) and depression (Beta = 0.09; 95% CI 0.02–0.16) after controlling for pre-pandemic anxiety and depression, respectively. French children with emotional difficulties or symptoms of hyperactivity/inattention reported more screen time (>1 h per day) during COVID-19 (32). It is worth noting that gaming/internet also predicted higher levels of externalizing behavior.

Although most included studies consistently found that there was an association between excessive screen time and children's mental health, Larsen et al. (54) found contradictory results. They did not find any association between the screen time and poor mental health outcomes in children.

##### Sleep disturbances

Of the 30 included studies, about one-third explored the association between sleep and children's mental health during COVID-19 (32, 43, 47, 48, 50, 55, 58, 61, 63, 67). With the closure of schools, home confinement and social isolation which led to significant changes in children's daily routines and activity patterns, children's sleeping patterns, such as sleep timing (delayed bedtime and rise time) and quality, were altered. Di Giorgio et al. (43) found that children's sleeping time has changed as they go to bed 53 min later and wake up about 66 min later than usual. Another study on 21,526 Chinese parents found that 74.7% parents said their children showed sleep disturbances in terms of bedtime resistance, sleep duration, sleep anxiety, sleep onset delay and night waking (55).

The quality of sleep impacted children's psychological wellbeing. Moulin El-Aarbaoui et al. (32) found that children's emotional difficulties and hyperactivity/inattention symptoms were significantly linked to sleeping difficulties. McArthur et al. (58) found that child sleep (Beta = −0.11; 95% CI −0.19−0.04) predicted anxiety. All the results point to the importance of sleep for children's psychological health.

##### Less physical activity

The existing literature supports the idea that physical activity is associated with many physical and mental health benefits across all age groups (69). Since children were forced to stay at home due to school closures and home confinement, the limited space at home as well as the restricted outdoor opportunities for children to be physically active were drastically reduced (70). Of the 30 included studies, eight studies (50, 55, 56, 58, 60, 61, 63, 67) examined the relations between physical activity and children's internalizing or externalizing behaviors. In an internet-based survey of 1,264 children, Liu et al. (56) found that when compared with children who did not do any physical activity, children with physical activity had a lower risk of hyperactivity-inattention (OR 0.44 for 1–2 days/week; OR 0.56 for more than 2 days/week) and fewer prosocial behavior problems (OR 0.65 for 1–2 days/week; OR 0.55 for more than 2 days/week). Overall, the results from the included studies consistently showed that there was a decrease in physical activity among children during the pandemic and a decrease in outdoor playtime. This substantial impact on the level of physical activity of children may affect children's physical and mental health (50, 63).

#### Unfavorable family environment factors

##### Increased COVID-19-related stressors among parents/primary caregivers

Of the 30 included studies, thirteen studies examined the association between perceived parental stress and children's emotional and behavioral problems during the pandemic (42, 46, 49, 51, 53, 54, 57, 61–66). Parents experienced mounting pandemic-related stress and COVID-19 contextual hardship (65), including an increase in childcare responsibilities, greater home-schooling demands and worries about the balance between increased caring responsibilities and work, family financial instability, fear of the future and many other difficulties, therefore they had higher levels of worries, anxiety and even parental burnout (a syndrome characterized by overwhelming exhaustion and the feeling of lacking achievements as parents) (51), which is detrimental to children's mental health, as children are particularly sensitive to the emotional status of their parents (71). Köhler-Dauner et al. (53) found that there was a positive and significant effect of maternal perceived stress on children's emotional problems and hyperactivity and inattention behaviors. Kerr and colleagues (51) utilized path modeling to analyze cross-sectional data from 1,000 parents and reported that parents who perceived more psychological impacts from the pandemic reported higher levels of parental burnout and less positive behavior in their children. Robertson et al. (62) investigated 286 linguistically, racially and ethnically diverse caregivers and found that caregivers' pandemic-related stress at Time 1 predicted increased child internalizing symptoms at Time 2 which, in turn, predicted increased caregivers' perceived stress at Time 3. The findings based on longitudinal data provided strong evidence for the reciprocal relationship between caregivers' perceived stress and children's mental health. The results further gave prominence to what is highlighted by the family system theorists - families are interconnected and mutually influenced (72).

Noteworthy, parents' negative affect may have spillover effects on their children *via* parenting (19, 73). The results of a large meta-analysis showed that parents' negative emotions were associated with harsher discipline whereas positive emotions were associated with more supportive parenting (74). Based on a cross-sectional study on 1,264 primary school children, Li and colleagues (55) found that Chinese parents reported exerting harsh parenting (i.e., scolded their child by yelling or shouting to discipline their children and regulate their misbehavior) during the pandemic (aOR 2.06; 95% CI 1.91–2.23). Harsh parenting was independently associated with child mental health problems, regardless of SES.

##### Deteriorated mental health of parents

Child mental health is closely linked to the wellbeing of the family members (58). This fact is supported by many existing studies, which provide strong empirical evidence that maternal wellbeing predicts child wellbeing (75, 76). About one-fifth of the included studies examined how parents' mental health impacted children's internalizing and externalizing behaviors (32, 39, 44, 45, 55, 56, 66, 67). Wang et al. (67) stressed that better parental wellbeing was associated with a decrease in child mental health problems. Liu et al. (56) found that children with anxious parents were associated with higher levels of emotional symptoms (OR 5.64, 95% CI 2.18–14.58) and total difficulty (i.e., emotional symptoms, conduct problems, hyperactivity/inattention and peer relationship problems) (OR 3.78, 95% CI 1.56–9.15) than children without anxious parents. The findings from the multiple regression analyses showed that more parental depressive symptoms predicted children's internalizing behaviors during the lockdown (45).

The longitudinal data yielded similar results. Frigerio et al. (47) found that children's emotional and behavioral problems significantly increased from the period preceding the lockdown to the period during which the lockdown was taking place and greater maternal mood symptoms were associated with an increase in anxious-depressed, withdrawn and aggressive symptoms of children during the lockdown.

#### Protective factors for child mental health

##### Resilience of parents

COVID-19 is a good instance of traumatic stress which creates significant impacts on the parents' resilience to deal with many stressors outlined in the foregoing paragraphs. Of the 30 included studies, only two studies explored the relationship between parents' resilience and children's mental health (57, 65). In Mariani Wigley et al.'s study (57), mothers' resilience was positively correlated with children's resilience. The relationship between mothers' resilience and children's stress-related behaviors was mediated by the mothers' ability to support and promote children's resilient behaviors which influences children's positive adjustment in the face of stressful situations. Therefore, parents' resilience is vital to children's mental health.

##### Positive parent-child relationships

It is well documented that positive parent-child relationships, which are fostered by positive parenting and supportive parental behavior, can mitigate the child negative outcomes from stressors (77). Some of the included studies examined the association between parent-child relationship and child mental health problems (39, 45, 46, 58). For instance, in Dubois-Comtois et al.'s study (45), closeness in the parent-child relationship was significantly negatively correlated with children's externalizing behaviors. Bate and colleagues (39) revealed that the emotional and behavioral health of children was moderated by positive parent-child relationships during the COVID-19 lockdown. McArthur et al. (58) found that child happiness during the COVID-19 pandemic was predicted by the connectedness to parents/caregivers (Beta = 0.36; 95% CI 0.28–0.39). In addition, lower levels of connectedness to parents/caregivers predicted child anxiety (Beta = −0.16; 95% CI −0.22 to −0.09) and depressive symptoms (Beta = −0.26; 95% CI −0.32 to −0.21) during COVID-19. Therefore, fostering positive parent-child relationships can help improve the mental health outcomes of children.

### School factors

#### School connectedness

Schools cannot alleviate harms caused by the pandemic (59). However, they can play a crucial role in supporting children through maintaining virtual contacts throughout lockdowns and school closures. Therefore, school connectedness is important to children. School connectedness has been defined in many ways, but it encompasses engaging students academically and in school activities, having a sense of belonging and fairness, developing positive peer relationships as well as feeling supported by teachers and feeling secure at school (78). In Moore et al.'s study (59), surprisingly, there were no changes in school connectedness before and after the pandemic. The participating children reported that they had a good teacher-student relationship which was significantly associated with better mental health and life satisfaction.

## Discussion

This systematic review aimed to identify the types of children at risk of developing mental health issues and summarize the risk and protective factors of children's mental health during the pandemic. Overall, the results of the 30 included studies consistently suggested that a negative impact of COVID-19 was observed on children's mental health who exhibited an increase in internalizing and externalizing behaviors. As the included studies were conducted in different countries, it is evident that children's mental health has become an area of concern globally.

Based on the 30 included studies, the worsened child mental health outcomes reflect socioeconomic inequalities. Parents with low education attainment tend to be employed in occupations that do not allow to work from home such as drivers, waiters/waitresses or supermarket workers. Their unstable income has been further affected by the pandemic resulting in either significantly reduced income or loss of employment. Financial insecurity is harmful to individuals' mental health as this can increase stress or anxiety which can worsen parenting practices leading to neglect or physical or verbal punishment or even abuse. Harsh parenting increases the risk of children's mental health problems. In addition, economically disadvantaged children normally live in crowded apartments. However, during the pandemic, housing has been a key determinant of health. Overcrowding and the size of private outdoor space play significant roles in how families adjust to social distancing restrictions and how to minimize the chance of contracting the virus from family members. It is not surprising that children living in cramped homes are more stressed (79). As Patel et al. (80) pointed out, “the pandemic has highlighted the stark inequalities within society, and it will likely exacerbate them” (p. 110). Now is therefore an opportune time for policy makers to introduce legislation to support those disadvantaged families.

Interestingly, being the only child in the family can be a risk factor. It is likely that larger household sizes mitigate some of the negative effects of social isolation, so the number of children in the household is protective of child mental health (81).

Using Bronfenbrenner's ecological systems theory (EST) (82, 83) as a lens enables us to better understand why the pandemic has had considerable impact on children's mental health. According to the EST, a child develops through the interaction of different systems (i.e., the micro-, meso-, exo-, macro- and chronosystems) from the closest to the broadest (84). During the pandemic, the children's microsystem has been compromised (38, 85) by a number of restrictions, such as the prolonged closure of schools, homeschooling and the absence of social support (peer relationships and extended families). All these persistent stressors, including a change in children's structured daily routine, increase the vulnerability of children. In addition, for parents, many pandemic-related stressors, such as juggling on-going work obligations with added childcare responsibilities and trouble in paying bills due to financial problems etc., may amplify any pre-existing mental health problems and result in higher levels of stress, anxiety and distress. If individual resilience is low, the negative psychological effects of stressors cannot be buffered. As a result, stressors may further deteriorate parents' mental health which can adversely affect family functioning resulting in problems such as more family conflicts and poor parent-child relationship. More importantly, due to the bidirectional relationship between parents and children in the microsystem, both parents and children are affected in a reciprocal manner.

During home confinement, the mesosystem has also been affected. With reduced support from collaborations between school and family (86), it is difficult for children to connect with teachers to seek support due to the closure of schools. However, it is vital for students to feel connected with schools as school connectedness has a buffering effect in helping mitigate negative mental health outcomes as shown in the current review (87).

Since both the micro- and mesosystem are embedded within the exosystem which is the layer that a child does not have direct interactions with, the exosystem impacts child development indirectly. For instance, many parents who worked remotely from home during home confinement experienced an increase in negative emotions which may have spillover effects on children indirectly *via* parenting practices.

The macrosystem can affect other systems as it includes political disturbances, cultural characteristics or economic disruption. During COVID-19, the shutdown measures have caused a substantial decline in the global economy. Some companies were forced to close permanently. Many people had to spend their emergency savings or borrowed loans to support their expenses, so parents have been under a heavy financial burden which is detrimental to their mental health.

The chronosystem, which incorporates the effect of time on individuals' development, includes both normative (e.g., graduation from school) and non-normative life transitions (e.g., parental divorce), environmental events and historical events. In our review, Moore et al. (59) found that there was no evidence that the pandemic had any consequences on children's feelings about the transition from primary to secondary school. The transition from primary to secondary education is a challenge and can be very stressful for children. During the pandemic, children were deprived of a well-planned transition by schools and families which can normally help remove barriers to learning and allow children to reach their full academic potential later on and not feel isolated. Future studies can explore the impact of the pandemic on children's transition in their schooling and psychological wellbeing.

Unhealthy lifestyle factors affect children's mental health. One of the root causes of unhealthy lifestyle is a lack of structured daily routines. Maintaining a structured and pre-planned day is a protective factor of children's mental health (88). Therefore, good and healthy daily habits for children can reduce the risk of mental health problems and improve their psychological wellbeing (89–91). Parents are encouraged to create and maintain healthy structured routines including sleep schedules and family media plans that foster the healthy use of recreational mobile devices (e.g., limits on duration). However, interventions focusing on reducing infants' and toddlers' screen time should be more targeted to parents, particularly parental mental health, screen time, intention to offer mobile devices to children and the needs of using mobile devices (92). Furthermore, since physical activity is associated with psychological health, promoting physical activities that can be performed in a limited space at home should be highly recommended to better support the psychological health of children.

Overall, the included studies have shown the negative impacts of the pandemic on children's mental health through the interaction of different factors. We need to support children's mental health recovery from the pandemic and it is a public health priority which requires effective actions at multiple levels of the society. Since children are particularly vulnerable and need adequate parental support, it is essential to ensure parents' mental health remains good, therefore the provision of more enhanced mental health resources and support programmes to parents to help them reduce stress, anxiety and depressive symptoms is urgently needed. With concerted efforts, children's lives can be improved.

### Implications for research

When reviewing all the studies, we observed that first, many empirical studies included both children and adolescents in the same study. The limitation is that the results generated from the studies were imprecise. Designing tailor-made interventions for children becomes more difficult as children and adolescents are at different developmental stages, so future studies on children's mental health are recommended. Second, studies define the age range of children differently, so this makes the comparison of findings difficult. Third, children's data were mostly collected from mothers' reports as they are generally the primary caregivers for children. Although the literature supports this method, it is essential to take into consideration not only mothers but also other caregivers, particularly fathers whose perspectives are limited in the existing literature. Fourth, future research is needed to investigate the longitudinal impact of maternal and paternal mental health on child development due to the COVID-19 pandemic, taking into account multiple time points instead of only two. In addition, since the majority of included studies are quantitative studies, future studies could use mixed methods or longitudinal qualitative studies to capture the experience and impact of the pandemic on children's mental health over time.

## Limitations of the review

The present systematic review has several limitations. First, the articles were retrieved from two large electronic databases and hand searched. Only those consisting of the relevant search terms in the title or abstract were reviewed for further analysis. Therefore, the selection of reviewed studies was limited. Second, studies that were not in English, published in conference abstracts, letters, government reports, textbooks and unpublished dissertations were excluded. Third, children with special education needs are excluded in the selection so the mental health of this group of children is less known.

## Conclusion

The systematic review summarized important information about children who are at risk of mental health problems in the context of COVID-19 as well as the risk and protective factors of children's mental health. The current review serves as a wake-up call to the government to provide targeted mental health care in the community to support children, especially those severely stressed by the pandemic. Children's mental health should be one of the top priorities of the post-pandemic recovery plan.

## Data availability statement

The original contributions presented in the study are included in the article/Supplementary material, further inquiries can be directed to the corresponding author.

## Author contributions

CN contributed to the design of the search strategy, conducted the initial searches, analysis of the findings, and prepared the first draft of the manuscript. SN conducted updated searches, reviewed the articles, and conducted the analysis of the findings. Both authors contributed to the revisions of the manuscript and approved the submitted version.

## Conflict of interest

The authors declare that the research was conducted in the absence of any commercial or financial relationships that could be construed as a potential conflict of interest.

## Publisher's note

All claims expressed in this article are solely those of the authors and do not necessarily represent those of their affiliated organizations, or those of the publisher, the editors and the reviewers. Any product that may be evaluated in this article, or claim that may be made by its manufacturer, is not guaranteed or endorsed by the publisher.
