# Supplementary material for: Impact of the COVID-19 pandemic on children's mental health: A systematic review
Source: Front Psychiatry. 2022 Oct 18;13:975936. doi: 10.3389/fpsyt.2022.975936 (PMC9622998; doi:10.3389/fpsyt.2022.975936)
Supplement: Supplementary file 1 [file Table_1.pdf]

Table A1. Results of the Quality Assessment for Included Studies

Study No	Included articles/Reference no.	Methodological items for rating (1-4)									
		Abstract & Title	Introduction & Aims	Method & Data	Sampling	Data Analysis	Ethics & Bias	Results	Transfer-ability	Implication & Usefulness	Total Score
**1**	Bate et al. (39)	4	4	4	3	4	3	4	3	3	32
**2**	Bhogal et al. (40)	4	4	3	2	4	2	4	2	3	28
**3**	Browne et al. (41)	3	3	2	3	3	3	3	3	3	26
**4**	Christner et al. (42)	4	4	4	2	4	3	4	3	4	32
**5**	Di Giorgio et al. (43)	4	3	4	3	4	3	4	3	4	32
**6**	Dollberg et al. (44)	4	4	3	2	4	4	4	3	4	32
**7**	Dubois-Comtois et al. (45)	4	4	4	3	4	3	4	3	4	33
**8**	Foley et al. (46)	4	4	3	3	3	3	3	3	3	29
**9**	Frigerio et al. (47)	4	4	3	2	3	3	3	2	2	26
**10**	Gassman-Pines et al. (48)	3	3	3	2	3	2	3	2	3	24
**11**	Giannotti et al. (49)	4	4	4	2	4	3	4	3	4	32
**12**	Hyunshik et al. (50)	3	4	3	3	3	4	3	3	3	29
**13**	Kerr et al. (51)	4	4	3	4	4	3	4	3	4	33
**14**	Köhler-Dauner et al. (52)	4	4	3	2	4	2	4	2	3	28
**15**	Köhler-Dauner et al. (53)	4	4	4	2	4	3	4	2	4	31
**16**	Larsen et al. (54)	4	4	3	2	3	3	4	2	3	28
**17**	Li et al. (55)	4	4	4	3	4	4	4	3	4	34
**18**	Liu et al. (56)	4	4	3	3	3	3	3	3	3	29
**19**	Mariani Wigley et al. (57)	4	4	3	3	4	3	4	2	4	31
**20**	McArthur et al. (58)	4	4	3	3	4	3	4	3	4	32
**21**	Moore et al. (59)	4	4	3	3	4	3	4	3	4	32
**22**	Moulin et al. (32)	4	3	3	4	4	4	4	4	4	34
**23**	Oliveira et al. (60)	4	4	2	2	3	2	3	2	3	25
**24**	Park et al. (61)	3	2	2	3	2	3	2	3	3	23
**25**	Robertson et al. (62)	4	4	3	3	3	3	4	3	3	30
**26**	Sama et al. (63)	2	2	1	2	2	1	2	2	1	15

		Methodological items for rating (1-4)									
**Study No**	**Included articles/Reference no.**	**Abstract & Title**	**Introduction & Aims**	**Method & Data**	**Sampling**	**Data Analysis**	**Ethics & Bias**	**Results**	**Transfer-ability**	**Implication & Usefulness**	**Total Score**
**27**	Specht et al. (64)	3	3	3	1	2	4	2	1	2	21
**28**	Thompson et al. (65)	4	4	3	3	4	3	4	3	4	32
**29**	Wang et al. (66)	4	4	3	3	4	4	4	3	4	33
**30**	Wang et al. (67)	4	4	4	3	4	3	4	3	4	33
